# Surgical Management of Dentin Hypersensitivity Associated With Noncarious Cervical Lesions in a Patient With a Psychiatric History: A Case Report

**DOI:** 10.7759/cureus.98050

**Published:** 2025-11-28

**Authors:** Akira Hausike, Shuichi Sato

**Affiliations:** 1 Department of Periodontology, Nihon University School of Dentistry, Tokyo, JPN

**Keywords:** atypical psychosis, cold sensitivity, connective tissue graft, hypersensitivity, periodontal surgery, psychiatric comorbidity

## Abstract

Oral and mental health have a linked and complex relationship. We report the case of a 47-year-old female with severe cold sensitivity localized to the upper right premolar region, who was unresponsive to desensitizing agents, behavioral interventions, and brushing modifications. Her psychiatric history included atypical psychosis requiring hospitalization, and she remained stable on antipsychotic medication at the time of the dental consultation. Despite conservative management, hypersensitivity persisted in the regions with significant wedge-shaped defects and gingival recession. Periodontal surgical intervention with a coronally advanced flap and subepithelial connective tissue graft was performed, resulting in complete and sustained resolution of symptoms at four months post-surgery. Beyond local symptom control, the patient also reported reduced anxiety associated with oral discomfort. The elimination of cold-induced pain during meals and tooth brushing decreased somatosensory preoccupation and improved daily functioning. Although no change in psychotropic medication dosage was made - specifically, brexpiprazole (1 mg daily), quetiapine (25 mg daily), and clonazepam (0.5 mg daily, with an additional as-needed dose permitted, maximum once daily) - her psychiatrist noted stable affect with less focus on somatic distress. While anecdotal and based on patient self-report, these observations suggest that alleviating persistent orofacial symptoms may contribute to improved psychological well-being in medically complex patients. This case illustrates that with interdisciplinary communication and medical stabilization, psychiatric disorders need not preclude periodontal plastic surgery and that individualized, coordinated management can extend the indications of root coverage procedures to include the treatment of therapy-resistant hypersensitivity.

## Introduction

Dentin hypersensitivity (DHS) is defined as a short, sharp pain arising from the exposed dentin in response to external stimuli such as thermal, tactile, or osmotic triggers that cannot be ascribed to any other dental pathology [1]. The hydrodynamic theory remains the most widely accepted explanation, proposing that fluid movement within open dentinal tubules activates the pulpal sensory nerves, ultimately eliciting pain. While this model accounts for the final pathway of pain induction, the preceding processes leading to hypersensitivity vary greatly among individuals and involve a complex interplay of biological, behavioral, and psychosocial factors. Recent reviews have emphasized that this multifactorial nature requires a tailored therapeutic approach that integrates both desensitizing and surgical strategies, depending on the lesion depth and patient background [2].

Recent studies have focused on the intricate connection between oral and mental health [3]. Patients with psychiatric disorders frequently present with oral conditions influenced by impaired self-care, side effects of medication, or parafunctional habits such as bruxism. Moreover, altered pain perception and anxiety regarding treatment may complicate the diagnosis and management of dental pain, including DHS. These factors often make clinical care challenging, especially when behavioral or psychosomatic components overlap with localized dental pathologies.

In most previously reported cases, periodontal plastic surgery for root coverage has been performed primarily for esthetic reasons or to improve the long-term prognosis of teeth affected by gingival recession. Only a few reports have described surgical interventions aimed at alleviating dentin hypersensitivity. In patients with psychiatric disorders, performing periodontal surgery poses additional difficulties, not only because of altered pain perception and psychological instability, but also because of potential challenges in ensuring postoperative compliance and maintenance. Consequently, the surgical management of DHS in such patients has rarely been attempted and documented.

We describe a patient diagnosed with atypical psychosis who presented with localized dentin hypersensitivity associated with noncarious cervical lesions (NCCLs) [4]. This report explores the multifactorial etiology of the condition and details a stepwise treatment approach that included initial management with topical desensitizing agents, behavioral modification, and occlusal adjustment, followed by periodontal surgical intervention when these conservative measures failed to achieve symptom relief. This case underscores the importance of individualized, interdisciplinary care and illustrates that when appropriately managed, surgical periodontal treatment can be both feasible and effective, even in patients with psychiatric vulnerabilities.

## Case presentation

Initial presentation and diagnosis

A 47-year-old woman presented with a chief complaint of severe localized cold-induced pain in the upper right premolar region.

Psychiatric history

The patient had a complex psychiatric history beginning in 2005, when she developed recurrent episodes of dizziness, abdominal discomfort, and diarrhea associated with occupational stress. She received care at several psychiatric institutions and was diagnosed with postpartum psychosis following the birth of her first child in 2011, which was successfully managed with medication. In 2022, she experienced worsening anxiety and somatic symptoms, including excessive concern about the potential adverse effects of the COVID-19 vaccination. Her condition progressed to include disorganized speech, frequent psychogenic seizures, and paranoid ideation, particularly the belief that the vaccine caused permanent harm. She was eventually hospitalized under medical protection because of acute psychiatric deterioration and suicidal behavior. Physical restraint was required during the early phase of hospitalization. The patient was diagnosed with atypical psychosis and treated primarily with aripiprazole (6 mg/day). Her symptoms gradually improved, and she was discharged in December 2022.

In 2024, she experienced recurrent anxiety and panic episodes, prompting the resumption of outpatient psychiatric care. Owing to aripiprazole-induced akathisia, the regimen was switched to brexpiprazole (1 mg/day), which achieved stable symptom control. As of April 2025, her psychiatric condition remained stable during regular follow-up. A concurrent psychiatric consultation confirmed her overall stability but noted a tendency toward heightened somatic sensitivity. Her psychiatric medications at the time of dental presentation were brexpiprazole (1 mg daily), quetiapine (25 mg daily), and clonazepam (0.5 mg daily), with an additional as-needed dose permitted (maximum once daily). She was a former smoker, had quit three years earlier, and was otherwise systemically healthy, aside from her psychiatric condition.

Dental findings

At the time of dental evaluation at a private clinic, the patient complained of severe localized dental pain triggered by cold stimuli that markedly interfered with eating, tooth brushing, and overall daily comfort. This symptom was new-onset and had not been reported during prior psychiatric visits. Her dentist referred her to a dental hospital for specialist assessment, where a periodontist assumed responsibility for her care.

**Figure 1:**
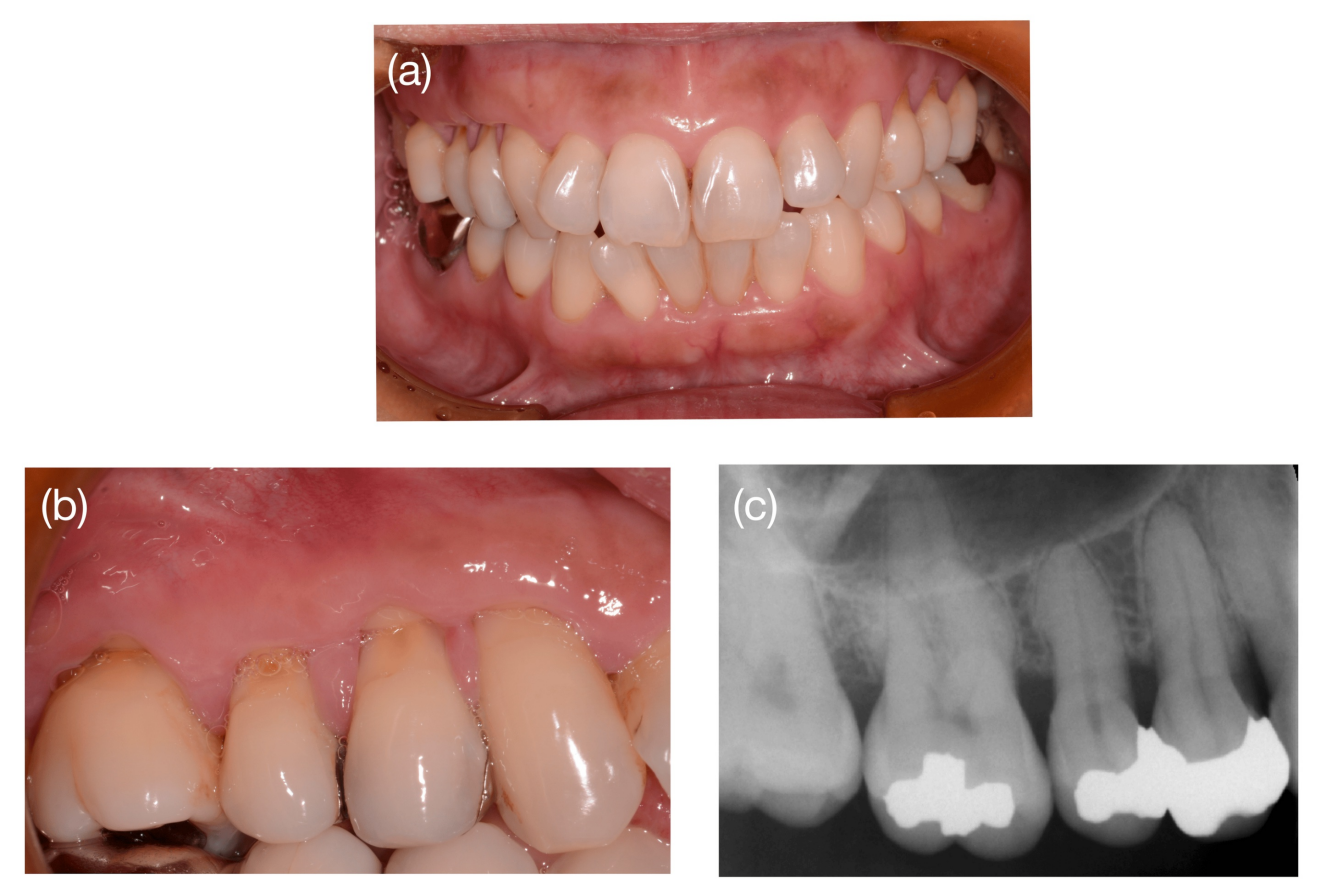
Initial views captured in a 47-year-old woman a) Facial view. b) Severe noncarious cervical lesions (NCCLs) were observed in the right maxillary region. c) Only mild bone resorption was confirmed, and no remarkable findings were noted on the radiograph.

Clinical examination revealed bilateral localized DHS in the upper premolar regions with severe symptoms on the right side. She rated her cold-induced pain as 8 on the visual analog scale. In the right maxillary region, deep wedge-shaped NCCLs were observed at the buccal cemento-enamel junction (CEJ) of teeth #13-#16, showing abrasion of both the enamel and cementum. Gingival recession was also observed in the same area (Figure 1). Because the CEJ line was indistinct owing to abrasion, the exact recession depth could not be directly measured; therefore, the gingival recession (GR) was calculated based on an estimated ideal CEJ line. The initial clinical parameters of teeth #13-#16 are summarized in Table 1. The maxillary right premolar region was diagnosed as Miller class I/recession type 2 [5,6].

**Table 1:** Clinical parameters at baseline and postoperative follow-up. * The most apical extent of the composite resin restoration as a reference point REC, recession; PPD, probing pocket depth

Labial site		Tooth #16			Tooth #15			Tooth #14			Tooth #13		
		Distal	Central	Mesial	Distal	Central	Mesial	Distal	Central	Mesial	Distal	Central	Mesial
Baseline	REC (mm)	4			5			6			3		
	PPD (mm)	3	2	3	3	2	3	3	2	3	2	2	3
Prior Surgery	REC (mm)	3			3*			5*			2		
	PPD (mm)	3	2	3	3	2	2	2	2	2	2	2	2
Follow-up	REC (mm)	1			0*			0*			0		
	PPD (mm)	3	2	3	2	2	2	2	2	2	2	2	2

To explore the possible etiologic factors, the patients’ occlusal and brushing habits were reviewed. The patient reported a history of clenching during both day and night, although she noted that this behavior had recently subsided. Regarding brushing habits, she demonstrated a palm grasp and applied excessive force.

Initial treatments

Although the fabrication of an oral appliance is recommended for occlusal force control, the patient declined. Instead, the patient was instructed to practice self-awareness techniques and consciously avoid tooth contact while awake. She was advised to switch to a pen grasp and use small, gentle brushing motions. To alleviate the symptoms, a topical desensitizing agent was applied to the buccal cervical regions of both the right and left premolars. To promote future gingival coverage and epithelial reattachment, a fluoroaluminocalcium silicate-based desensitizer, Nanoseal (Nippon Shika Yakuhin, Yamaguchi, Japan), was selected as the topical desensitizing agent and applied according to the standard protocol. This intervention led to symptom improvement on the left side; however, pain persisted in the upper right first premolar, where the wedge-shaped defect was deeper, and the gingival support was insufficient.

Surgical intervention for root coverage

**Figure 2:**
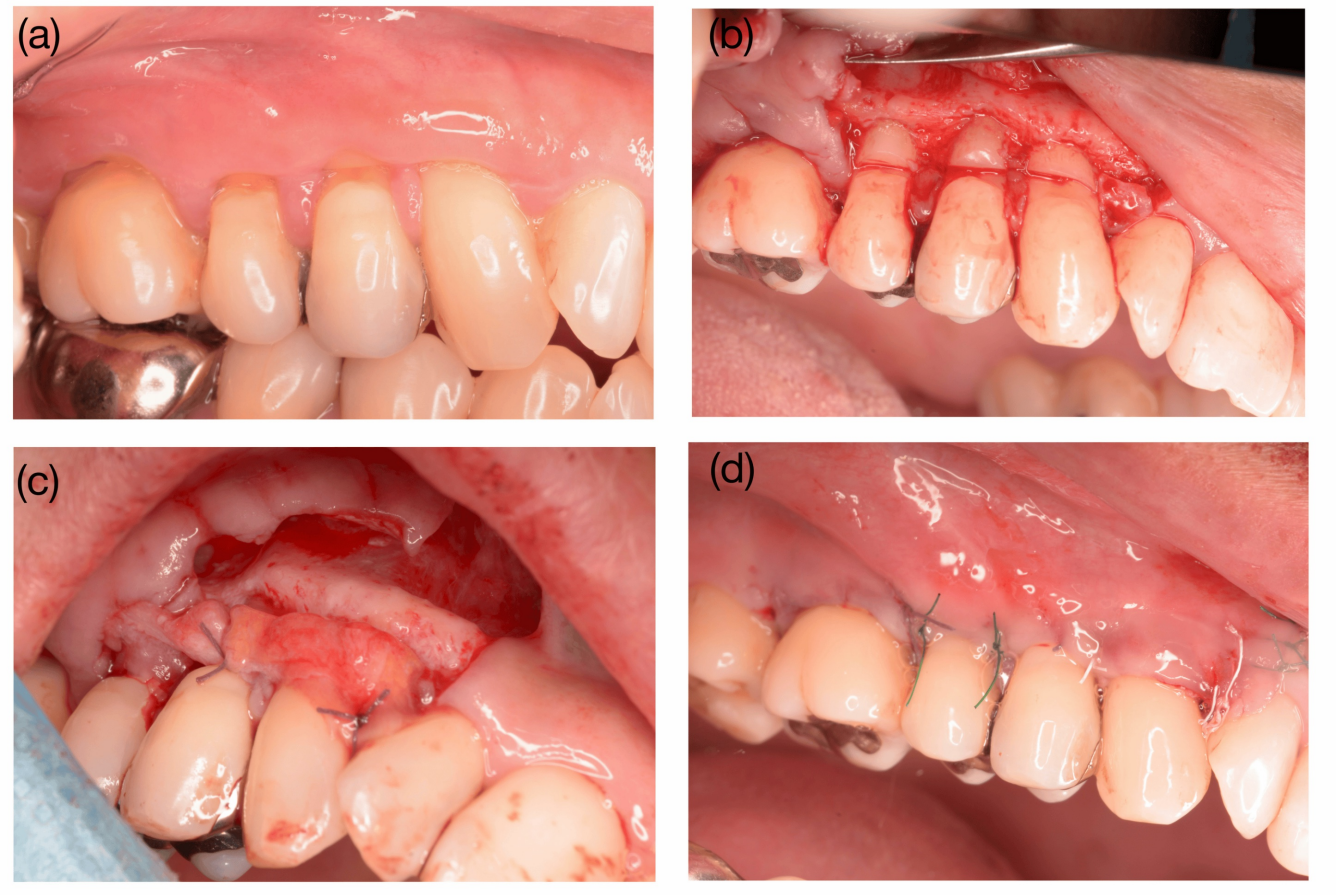
Surgical intervention in the right maxillary area. (a) Two weeks before surgery, a composite resin restoration was performed to restore the enamel component of the noncarious cervical lesions (NCCLs). (b) A single vertical releasing incision was placed distal to tooth #12 and extended beyond the mucogingival junction (MGJ). A horizontal incision was then made to create an envelope-type flap from #13 to #16, forming surgical papillae between #15–#14, #14–#13, and #13–#12. A full-thickness flap was elevated from #13 to #16. (c) After a deep horizontal releasing incision at the inner aspect of the MGJ, a connective tissue graft (CTG) harvested from the palatal premolar area was secured to the surgical papillae. (d) The flap was repositioned and stabilized with simple interrupted sutures.

Because the patient continued to experience hypersensitivity and expressed a strong motivation for definitive resolution, periodontal plastic surgery was performed in the right maxillary premolar region. Prior to surgery, the CEJ was virtually reconstructed using the contralateral teeth as references [7]. The coronal enamel portion of the NCCL was restored using a resin composite (Clearfil Majesty, Kuraray Noritake, Tokyo, Japan) to reestablish a smooth cervical contour and provide a stable reference for flap positioning (Figure 2a). The apical root surface, which originally contained cementum, was intentionally left unrestored to allow direct connective tissue attachment. The clinical parameters for teeth #13-#16 after restoration are summarized in Table 1, in which recession was recorded using the most apical extent of the composite resin restoration as the reference point.

Two weeks after resin composite restoration, the GRs were treated surgically. Following local anesthesia, a single vertical release incision was made in the distal part of tooth #12, extending beyond the mucogingival junction (MGJ). A horizontal incision was then made to design an envelope-type flap extending from #13 to #16, creating surgical papillae between #15 and #14, #14 and #13, and #13 and #12 (Figure 2b). The epithelial tissue of the surgical papillae was carefully removed using a scalpel. The design of these surgical papillae is crucial for achieving coronal advancement and preserving flap vascular integrity [8,9]. Additionally, an interdental tunnel was created between #16 and #15. A full-thickness flap was elevated from #13 to #16, followed by a deep horizontal release incision in the inner aspect of the MGJ to facilitate the coronal advancement of the flap.

A connective tissue graft (CTG) approximately 1 mm thick was harvested from the palatal premolar region as a free gingival graft and was extraorally de-epithelialized. The CTG was inserted beneath the flap and positioned under a nonincised anatomical papillary tunnel between #16 and #15 (Figure 2c). The graft was first secured just apical to the anatomical papilla between #16 and #15 using a simple interrupted suture with 5-0 resorbable suture material (5-0 Vicryl, Ethicon, Somerville, NJ, USA). Additional stabilization was achieved with simple interrupted resorbable sutures (5-0 Vicryl; Ethicon, Somerville, NJ, USA). The flap was then coronally advanced and stabilized with simple interrupted sutures engaging the de-epithelialized surgical papilla, complemented by sling sutures extending from the incised papilla to the tunneled papilla and to the papilla of the adjacent tooth not involved in the flap (5-0 Vicryl; Ethicon, Somerville, NJ, USA) (Figure 2d). The vertical incision was approximated to the adjacent soft tissue using simple interrupted sutures (GC Softretch #5-0, GC, Tokyo, Japan); (GC BioSoftretch #5-0, GC, Tokyo, Japan).

Shortly after the surgery, the patient received loxoprofen sodium 60 mg for pain relief. Postoperatively, the patient was instructed to avoid tooth brushing at the surgical site for 2 weeks. Loxoprofen sodium 60 mg was administered immediately after surgery and 6 h later. Sutures were removed on day 10, following which the patient began gentle brushing with a soft postoperative toothbrush. Professional prophylaxis was provided at weeks 2, 4, 6, and 12.

Follow-up

**Figure 3:**
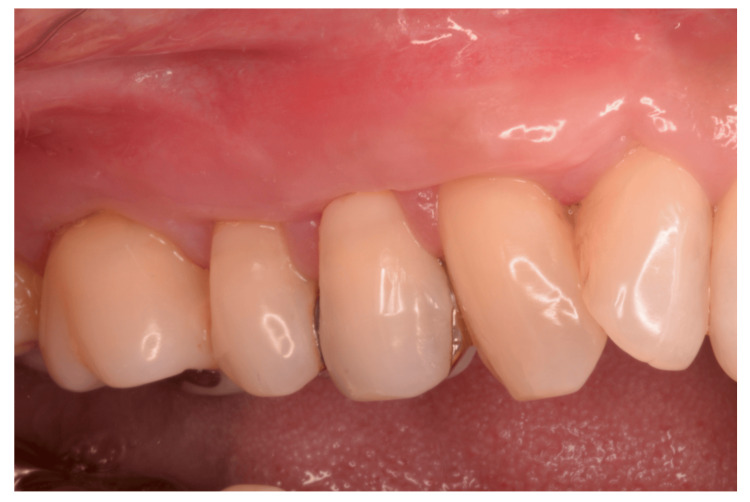
Clinical photograph at four months post-surgery. Complete root coverage is observed, and the patient reports no dentin hypersensitivity (DHS). Although a slight step in the gingival margin remains, the patient is satisfied with the overall outcome.

Healing progressed uneventfully. At the four-month re-evaluation, complete root coverage was achieved, and the patient reported complete resolution of DHS. Although a minor step remained at the gingival margin, the patient was highly satisfied with the overall outcome (Figure 3). The clinical examination results are summarized in Table 1. The probing pocket depths remained consistently shallow from the initial visit, and gingival recession, which measured up to 6 mm preoperatively, was reduced to nearly zero.

## Discussion

This case report demonstrates the successful management of severe DHS associated with NCCLs in a patient with psychiatric comorbidities, highlighting the complex interplay between mechanical, pharmacological, and behavioral factors. Furthermore, this illustrates that surgical intervention using CTG and a coronally advanced flap (CAF) can effectively control severe DHS accompanied by NCCLs.

**Figure 4:**
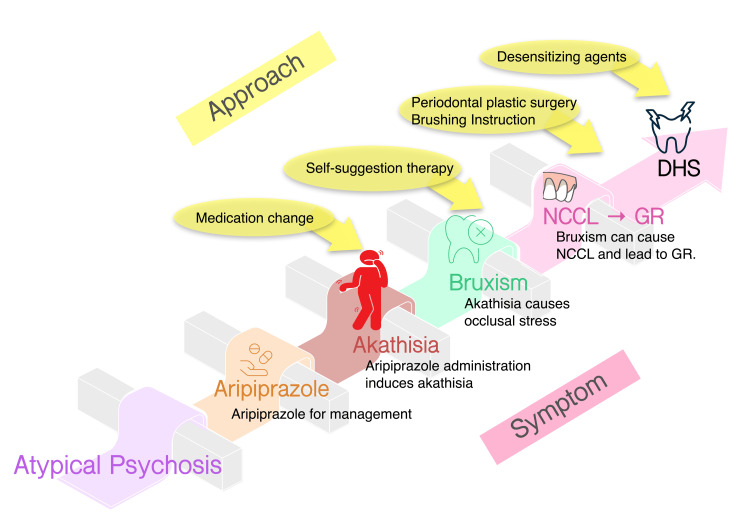
A proposed hypothesis linking atypical psychosis to DHS through akathisia and bruxism. Possible symptoms and approaches, including medication change, behavioral therapy, and dental management, are illustrated. NCCL, non-carious cervical lesion; GR, Gingival recession; DHS, Dentin hypersensitivity. Source: Authors.

The patient’s psychiatric profile adds an additional dimension to this multifactorial process, extending beyond simple orofacial disorders. At the first dental visit, the patient reported a history of Guillain-Barré syndrome in 2022 following the COVID-19 vaccination. Although an association between Guillain-Barré syndrome and COVID-19 vaccination has been described [10], no influence on dental diseases has been anticipated. However, through discussions with her psychiatrist, a plausible etiological link between psychiatric pharmacotherapy and DHS was hypothesized. This relationship remains hypothetical rather than causally established, as illustrated in Figure 4.

The patient reported a history of clenching during both the day and night, although she noted that this behavior had recently subsided after changing medication from aripiprazole to brexpiprazole, likely showing an etiological link between psychiatric pharmacotherapy and clenching. Atypical antipsychotic therapy with aripiprazole induces akathisia, a movement disorder characterized by restlessness and involuntary muscular activity [11,12]. Dopamine receptor-blocking or partial agonists such as aripiprazole are known to produce extrapyramidal side effects, including akathisia and bruxism [13]. A clinical case report described new-onset nocturnal bruxism in a 10-year-old boy temporarily associated with aripiprazole therapy, confirmed by symptom recurrence upon rechallenge, suggesting a probable adverse drug reaction according to the Naranjo scale [14]. This observation supports the potential of atypical antipsychotics to induce involuntary jaw movements through dopaminergic modulation, reinforcing the concept of drug-induced mechanical loading on the dentition.

Compared with aripiprazole, brexpiprazole exhibits lower intrinsic activity at D₂ receptors and has been associated with markedly reduced incidence of akathisia and other extrapyramidal symptoms, providing a pharmacological rationale for its better tolerability in long-term use [15]. Although a formal diagnosis of sleep bruxism has not been established, the improvement in clenching behavior after switching to brexpiprazole suggests a possible drug-related mechanism. Recent experimental evidence also indicates that individuals with probable sleep bruxism exhibit lower general pain tolerance and heightened subjective dental sensibility than nonbruxers, supporting the concept of somatosensory amplification that may contribute to exaggerated dentin sensitivity [16].

Chronic mechanical loading may contribute to the development of NCCLs and secondary GR, ultimately resulting in the development of DHS. In addition to occlusal stress and parafunctional habits, excessively forceful tooth brushing has also been recognized as an important etiological factor leading to cervical abrasion and dentin exposure. Further clinical and experimental studies are warranted to validate and refine the proposed etiological model.

Conservative treatment for NCCLs, including resin-based restorations and topical desensitizers, remains the first-line approach; however, the long-term effectiveness is limited when lesions extend beyond superficial dentin exposure. A fluoroaluminocalcium silicate-based desensitizer (Nanoseal) was selected instead of a resin-based system because in vitro studies have demonstrated its superior physicochemical stability in exposed root dentin. This material forms an acid-resistant, mineral-rich surface layer and promotes dentin remineralization through calcium and silicate ion release, effectively occluding the dentinal tubules, while avoiding polymeric residues that may interfere with subsequent soft tissue healing [17]. Recent scoping reviews have highlighted the importance of combining restorative and surgical procedures for deep NCCLs to achieve durable symptom control [18]. Surgical root coverage provides biological and mechanical protection in refractory cases. Meta-analyses have demonstrated that CAF combined with CTG provides superior long-term outcomes in reducing hypersensitivity compared with CAF alone or collagen matrices [19]. In the present patient, complete symptom relief within several weeks supports these findings, suggesting that increased gingival thickness and reestablishment of a tight mucogingival seal are critical determinants of durable pain control.

The restorative surgical protocol used in this case followed a biologically oriented approach suitable for combined NCCL-GR defects. Coronal reconstruction of the NCCL at the CEJ using composite resin improved flap adaptation and provided an accurate coronal reference for suturing [7]. The apical root surface was intentionally left unrestored to permit new epithelial and connective tissue attachment, thereby enhancing long-term stability. This integrative approach successfully restored both the anatomic contour and functional barrier against external stimuli.

In addition to the technical aspects, this case underscores the importance of interdisciplinary coordination. Psychiatric disorders can affect pain perception, oral hygiene, and treatment compliance, whereas psychotropic medications may induce xerostomia or motor disturbances that increase mechanical stress. Once psychiatric stability is achieved and medical collaboration is established, periodontal plastic surgery can be performed safely and effectively. The integration of biologically sound surgical reconstruction with coordinated psychiatric care resulted in symptom relief and functional recovery. Beyond local symptom control, the patient also reported reduced anxiety associated with oral discomfort. The elimination of cold-induced pain during meals and tooth brushing decreased somatosensory preoccupation and improved daily functioning. Although no change in psychotropic medication dosage was made, her psychiatrist noted stable affect with less focus on somatic distress. While anecdotal and based on patient self-report, these observations suggest that alleviating persistent orofacial symptoms may contribute to improved psychological well-being in medically complex patients.

This case illustrates that with interdisciplinary communication and medical stabilization, psychiatric disorders need not preclude periodontal plastic surgery and that individualized, coordinated management can extend the indications of root coverage procedures to include the treatment of therapy-resistant hypersensitivity.

## Conclusions

This case report demonstrates that severe DHS associated with NCCLs can be successfully resolved using a biologically oriented surgical approach, even in patients with psychiatric comorbidities. The combination of CAF and CTG provides durable pain relief by restoring gingival coverage and re-establishing a protective soft tissue barrier.
